# Cellulose Acetate Incorporating Organically Functionalized CeO_2_ NPs: Efficient Materials for UV Filtering Applications

**DOI:** 10.3390/ma13132955

**Published:** 2020-07-01

**Authors:** Madalina Elena Culica, Andreea L. Chibac-Scutaru, Violeta Melinte, Sergiu Coseri

**Affiliations:** “Petru Poni” Institute of Macromolecular Chemistry of Romanian Academy, 41 A, Grigore Ghica Voda Alley, 700487 Iasi, Romania; culica.madalina@icmpp.ro (M.E.C.); andreea.chibac@icmpp.ro (A.L.C.-S.)

**Keywords:** cellulose acetate, UV shielding, cerium, nanoparticle, materials

## Abstract

One of the major issues faced when constructing various materials incorporating inorganic nanoparticles (NPs) is aggregation leading to loss of their final activity. In our work, cellulose acetate (CA) has been used to serve as matrix for the synthesis of UV-shielding and transparent films containing various amounts (1–5 wt.%) of cerium oxide (CeO_2_) NPs. In order to attain an improved dispersion and a better connectivity between NPs and the cellulose matrix, the surface of CeO_2_ NPs have been previously functionalized by the reaction with 3-aminopropyl(diethoxy)methylsilane (APDMS). The uniform dispersion of the NPs in the homogeneous thin films has been evidenced by using Transmission Electron Microscopy (TEM) and Fourier Transformation Infrared Spectroscopy (FTIR) characterization. The investigation of the optical properties for the hybrid films through UV-Vis spectroscopy revealed that the presence of CeO_2_ NPs in the CA matrix determined the appearance of strong UV absorption bands in the region 312–317 nm, which supports their use as efficient UV absorbers. This study has shown that UV shielding ability of the nanocomposites can be easily tuned by adjusting the numberof inorganic NPs in the CA template.

## 1. Introduction

CeO_2_ has attained in the last years a lot of interest, as the most reactive rare earth material, being extensively used especially as catalysts due to its crucial importance in the design of three way catalysts (TWCs) and photocatalysts. This is due to the fact that CeO_2_ nanoparticles exhibit superior catalytic properties as compared to their macroscopic counterparts, mainly given by their special features such as large surface area and particular composition of surface defects and oxygen vacancies [1]. However, in the last years, several studies on the employment of ceria within new scientific areas related to biology and medicine have been reported, as for example, the utilization of CeO_2_ as a vehicle for intracellular drug delivery [2] or as a support for stem cells cultured in vitro [3]. CeO_2_ nanoparticles also proved that they could reduce ischemic brain deterioration by braking the blood–brain barrier after ischemia [4].

Besides the traditional use of CeO_2_ in catalytic and photocatalytic processes, CeO_2_ is envisaged to be implemented in a wide range of technical applications including material polishing, ultraviolet shielding, ceramics composites and sensors as it can easily absorb light in both visible and UV ranges [5]. Development of such applications requires the use of a convenient matrix for efficient incorporation of the nanoparticles, to enhance the performance, avoid the cluster growth and aggregation and improve the handling and recovery of the nanoparticles. Up to now, CeO_2_ particles were incorporated in various polymeric templates such as poly(meth)acrylates [6,7,8] polyurethanes [9,10], polyimides [11], polyamides [12] or polydimethylsiloxanes [13]. Among the plethora of polymers, one of the best candidates to immobilize CeO_2_nanoparticles (NPs), which fits both environmental and economic issues is represented by cellulose [14,15]. Cellulose, due to its intrinsic properties, like abundance, renewability, nontoxicity and biodegradability, combined with excellent mechanical properties is frequently studied, especially when taking into consideration that the plastic production is built mostly on feedstock extracted from oil refineries. It is not surprising that in the last decade we assisted with a tremendous upsurge in cellulose research attempting to replace the petroleum-based polymers in many applications. In a very recent review, cellulose is considered “a ubiquitous platform for ecofriendly metal nanoparticles preparation” [16] due to its versatility and efficiency to incorporate a wide range of metallic and metal oxide nanoparticles. Often, to impart a broader area of applicative domains, cellulose derivatives, including those carrying carboxyl groups as for example TEMPO-oxidized cellulosic materials in form of powder, fibers and films are prepared [17,18,19,20]. In this way, the newly introduced functionalities improve specific properties such as solubility in organic solvents and processability, also creating dedicated sites for anchoring various (bio)organic carriers. Another class of cellulose-based polymers, namely its esters, is commonly used in technical applications. One of the most common representatives of this class is cellulose acetate. This ester is usually obtained by cellulose esterification with acetic acid and acetic anhydride in the presence of sulfuric acid, followed by partial hydrolysis of the triacetate ester. Cellulose acetate, unlike cellulose has an excellent solubility in various organic solvents, having also a satisfactory mechanical strength, which allows its processing into films, membranes, or fibers [21,22]. Taking advantage of these features of cellulose acetate, it can be used for development of sustainable and environmentally friendly materials for the food packaging industry. For this purpose it is necessary to improve the poor UV light barrier properties of cellulose acetate films since UV light affects the food quality thus perturbing their storage for longer periods [23]. In this context, herein we would like to report the preparation of organic–inorganic hybrid films bearing functionalized CeO_2_ NPs, with UV-shielding capabilities. The use of CA, facilitates the experimental protocol, avoiding the complex solvent systems used previously for the regenerated cellulose–CeO_2_ NPs preparation [14]. Moreover, the film formation is a straightforward process, the only solvent employed being acetic acid. Before use, the CeO_2_ NPs were functionalized by reaction with 3-aminopropyl(diethoxy)methylsilane. In this way, –NH_2_ moieties are introduced on the NPs surface, which contribute to a better stabilization and attachment to the organic matrix. Additionally, the CeO_2_ NPs concentration can be manipulated, so that different grades of UV-shielding can be attained. The resulting films can also be combined using physical processes, like calendaring or hot pressing with various transparent films, opening wide alternatives of implementation in the field of UV protection.

## 2. Experimental

### 2.1. Materials

Cerium (IV) oxide nanopowder (<25 nm particle size (BET)), 3-aminopropyl(diethoxy)methylsilane, cellulose acetate (CA), M ~30,000 g/mol and 40 wt.% of acetyl, were purchased from Sigma Aldrich Chemical Co. (Taufkirchen, Germany) and used without further purification.

### 2.2. Preparation of CeO_2_-Functionalized Nanoparticles (CeO_2_–APDMS)

For the functionalization of cerium oxide nanoparticles, 2 g (11.6 mmol) CeO_2_ (previously dried in oven at 110 °C) was magnetically dispersed in 200 mL anhydrous toluene and then 7.5 mL (35 mmol) 3-aminopropyl(diethoxy)methylsilane was added. The reaction was carried out at reflux for 7 h under nitrogen atmosphere with vigorous stirring according to a procedure previously reported in the literature [24]. Then, the solvent was removed by centrifugation and the obtained nanoparticles were washed twice with toluene, ethanol and methylene chloride and were dried under vacuum for 48 h.

### 2.3. Synthesis of CeO_2_–APDMS/Cellulose Acetate Nanocomposite Films

CeO_2_–APDMS/CA films were synthesized by solution casting method. Thus, 1 g of CA was dissolved in 20 mL glacial acetic acid under continuous stirring until a homogenous solution was obtained. Then, a corresponding quantity of functionalized CeO_2_ nanoparticles (for the achieving of hybrid films containing 1, 3 and 5 wt.% CeO_2_ NPs), previously dispersed by sonication in glacial acetic acid for 3 h, was added to the CA solution and sonicated again for 1 h. The reaction mixture was transferred into a Petri dish and dried at room temperature until complete evaporation of the solvent, when flexible and homogeneous nanocomposite films were obtained.

### 2.4. Characterization

Fourier transform infrared (FTIR) spectra were registered on a Bruker Vertex 70 FT-IR spectrometer (BRUKER, Karlsruhe, Germany) using KBr pellets. Scanning electron microscopy (SEM) and energy-dispersive X-ray spectroscopy (EDX) analyses were obtained on an environmental scanning electron microscope QUANTA200 coupled with an energy-dispersive X-ray spectroscope (ESEM/EDX), FEI Company, Hillsboro, OR, USA, operating at 20 kV in low vacuum mode and using an LFD detector (Fei Company, Hillsboro, OR, USA). The SEM and EDX investigations were realized in cross section of the CA/CACe films and in different points of the sample to check their reproducibility. Transmission electron microscopy (TEM) images for CACe-1 were obtained on a FEI Titan CT microscope (FEI Company, Hillsboro, OR, USA) operating at 300 kV and TEM images for CACe-5 were obtained on a Tecnai Twin microscope (FEI Company, Hillsboro, OR, USA) operating at 120 kV. Before investigations, the cellulosic films were immersed in an epoxy resin and cured at 65 °C overnight, then thin sections of 80 nm were cut with an ultramicrotome, collected on a 300-mesh copper grid and imaged.

The UV-Vis absorption and transmittance spectra of the cellulosic films were measured using a Perkin Elmer Lambda 2 UV–Vis spectrophotometer (Perkin Elmer Inc., Wellesley, MA, USA) in the wavelength region of 200–800 nm. The photocatalytic activity of the films containing CeO_2_ NPs was evaluated following the behavior of aqueous phenol, Methyl Orange and Congo Red solutions under ambient conditions and UV irradiation. The experimental protocol was in an analogous manner to [25,26,27]: 100 mL of aqueous solution of phenol (10^−3^ M), Methyl Orange (5 × 10^−5^ M) or Congo Red (10^−5^ M) containing a piece of each hybrid film CACe-1/CACe-3/CACe-5 (1 g) was irradiated with UV light (Hg–Xe lamp, λ = 365 nm, light intensity ca. 10 mW/cm^2^) for 3 h. The pollutant solutions were analyzed before and after UV exposure using an UV-Vis spectrophotometer (Perkin Elmer Lambda 2).

The stress/strain curves were recorded on a Shimadzu AGS-J deformation apparatus (Shimadzu, Columbia, MD, USA) at ambient temperature and at a rate of deformation of 5 mm/min with a load cell capable of measuring forces up to 1 kN. For each sample, five tests were performed and the average value was taken into account. The Young’s modulus was evaluated from the slope of the stress–strain curve in the initial linear segment, while the toughness was calculated by integrating the area under the stress–strain curve. The thermal stability for the cellulose films was evaluated by thermogravimetric analysis (TGA) on a Thermogravimetric Analyzer, Discovery TGA 5500 (TA Instruments, New Castle, DE, USA) in the temperature range of 25–700 °C under a dry nitrogen atmosphere, at a heating rate of 10 °C min^−1^.

## 3. Results and Discussion

**Figure 1:**
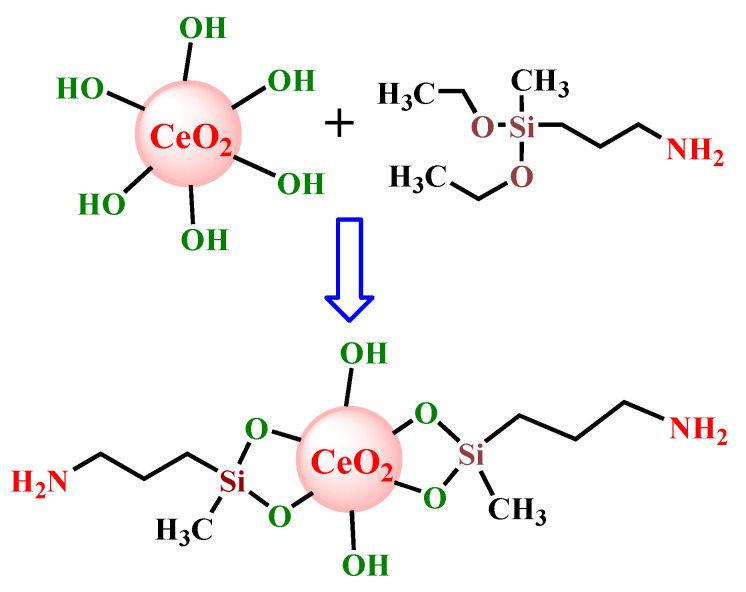
Schematic synthetic route for the functionalization of CeO_2_ nanoparticles (NPs) with amino units.

The homogeneous and complete distribution of the inorganic phase in the organic matrix in the case of hybrid composites favors the enhancement of the interfacial surface area, thus optimizing the organic–inorganic interactions responsible for the improvement of the final properties of the materials. However, this target is difficult to attain, especially due the inherent immiscibility between inorganic and organic phases, reason for that extensive research has been devoted to the chemical modification of inorganic nanoparticles [28]. The functionalization of CeO_2_ nanoparticles with silane sequences aimed to improve the compatibility between the inorganic units and the cellulose matrix with a direct effect on their better dispersion, reduced aggregation and stabilization in the organic material. The pathway used for the synthesis of CeO_2_ NPs functionalized with alkoxysilane groups is graphically illustrated in Figure 1.

**Figure 2:**
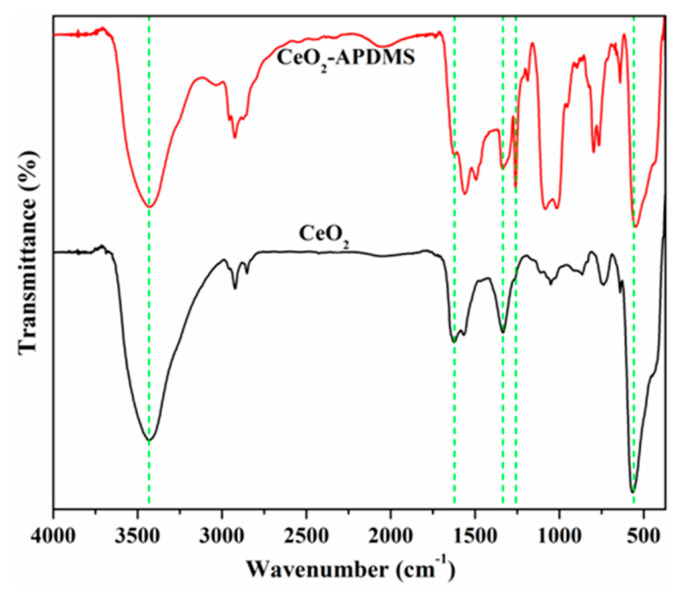
Fourier transform infrared (FTIR) spectra of CeO_2_ nanoparticles before (black) and after (red) silane functionalization.

To confirm the functionalization of CeO_2_ nanoparticles with 3-aminopropyl (diethoxy)methylsilane, the FTIR spectra of CeO_2_ and CeO_2_–APDMS nanoparticles were recorded and are represented in Figure 2. In the FTIR spectrum of CeO_2_ sample, the peak at 3433 cm^−1^ corresponds to –OH stretching vibrations of the hydroxyl groups, while in the modified sample, the –NH_2_ stretching of the primary amine is overlapping with the –OH stretching, which appears around the same frequency (3200–3600 cm^−1^) [29]. The absorption peaks located at 2957, 2924, and 2876 cm^−1^ corresponds to –CH_3_ and –CH_2_ asymmetric stretching vibrations. The band at 1624 cm^−1^ can be attributed to the interlayer stretching and bending vibration of molecular water. In the case of silylated nanoparticles (CeO_2_–APDMS), the absorption peaks at 1337 and 1261 cm^−1^ are characteristic of Si–CH_3_ and Si–CH_2_–stretching vibrations, while the strong absorption band at 1082 and 1016 cm^−1^ is caused by the Si–O–Si vibration of the grafted groups, confirming the modification of the surface of CeO_2_ NPs by silylation reaction [30]. The absorption band at about 555 cm^−1^ presented in both samples is representative of Ce–O stretching vibration [31].

**Figure 3:**
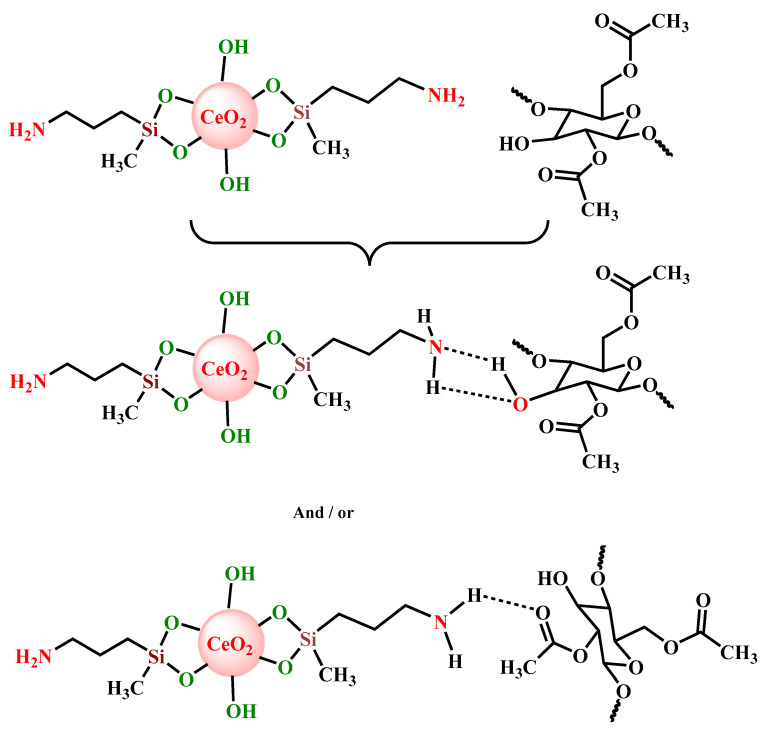
Possible interaction mechanism between CeO_2_–3-aminopropyl (diethoxy) methylsilane (APDMS) nanoparticles and cellulose acetate molecules.

Functionalized CeO_2_ NPs were further incorporated (in various quantities) in cellulose acetate films, in order to attain through a simple and efficient process, transparent flexible UV-protective coatings. The interaction between the –NH_2_ units from the surface of functionalized CeO_2_ NPs and cellulose acetate can be achieved by means of –OH or –C=O functionalities from the cellulose derivative (Figure 3), ensuring a suitable bonding among the organic matrix and inorganic component and a minimal leaching of the nanoparticles.

**Figure 4:**
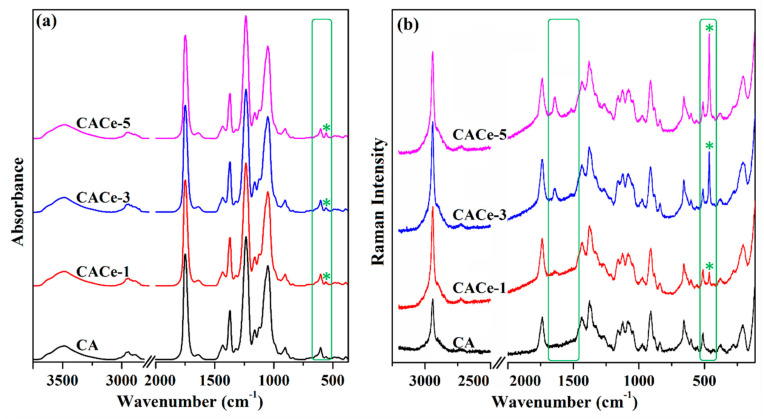
FTIR (**a**) and Raman (**b**) spectra of cellulose acetate and of the hybrid films CACe-1, CACe-3 and CACe-5 (* indicates Ce–O stretching vibration).

The FTIR spectra of the pristine cellulose acetate film and of the hybrid samples, registered in the 4000–370 cm^−1^ range (Figure 4a) were used to evaluate the structural information of the analyzed samples. As can be seen in the figure, the peaks characteristic to cellulose acetate are clearly visible at 3485 cm^−1^ (stretching vibration of O–H groups), 2889 and 2943 cm^−1^ (C–H stretching vibration), 1749 cm^−1^ (C=O stretching vibration), 1433 and 1369 cm^−1^ (C–H deformation), and 1234 and 1051 cm^−1^ (C–O stretching vibration) [32]. A small contribution that can be attributed to Ce–O stretching vibration can be noticed at 557 cm^−1^ in the FTIR spectra of the hybrid composites. Clearer differences between the analyzed samples appear in the Raman spectra (Figure 4b), where besides the characteristic peaks for cellulose acetate molecules, a new peak grows progressively with the number of CeO_2_ nanoparticles at 436 cm^−1^ and can be attributed to the F_2g_ Raman active-mode of ceria nanoparticles in a fluorite type cubic structure [31], while the band at 1636 cm^−1^ that gradually rises can be assigned to the intermolecular hydrogen bond development in the form of N–H…O=C interactions [33].

### 3.1. Structural and Morphological Characterization of the Materials

**Figure 5:**
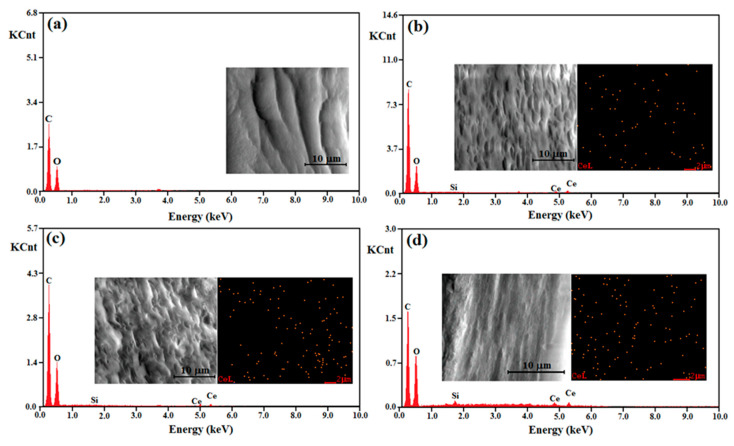
Energy-dispersive X-ray (EDX) spectra of cellulose acetate (CA) (**a**), CACe-1 (**b**), CACe-3 (**c**) and CACe-5 (**d**) films recorded in cross section. The inset shows SEM images and mappings of cerium in the film cross sections.

Complementary information about the chemical composition of the cellulose acetate-based films was achieved through energy-dispersive X-ray spectroscopy (EDX) on randomly selected areas of the cross sections of the films. In addition, the spatial distribution of cerium element in the cross sections of the hybrid materials was investigated through the X-ray elemental mapping images. In the EDX pattern of pure CA film, given in Figure 5a, the major elements observed were carbon and oxygen, meanwhile the spectra of the hybrid films containing functionalized CeO_2_ NPs (Figure 5b–d) presented also the signals specific for cerium and silicon elements, which confirmed that the functionalized CeO_2_ NPs were successfully loaded in the CA films.

For the CACe-5 film, the relative intensity of the Ce and Si signals was higher than in the case of the other two composites, being in good agreement with the bigger CeO_2_ NPs content incorporated in the cellulosic matrix. Moreover, the elemental mapping images of cerium atoms for the cellulose–CeO_2_ films (Figure 5b–d—inset, right side), suggested a relatively uniform distribution of the inorganic nanoparticles within the organic matrix.

The morphologies of the prepared films (CA and CACe-1-5) were investigated using SEM technique, the images of the cross-sections of the cellulosic films are displayed in the inset of Figure 5. The CA film had compact and uniform morphology, without cracks. The CACe-1–5 hybrids also presented a compact and uniform morphology, but the increase in the samples’ roughness was also noticed. Since the CeO_2_ NPs sizes are under 100 nm, their presence could not be detected by the SEM method for the achieved images at 10000× magnification; this also meant that the inorganic components were very well incorporated and dispersed in the cellulosic material.

**Figure 6:**
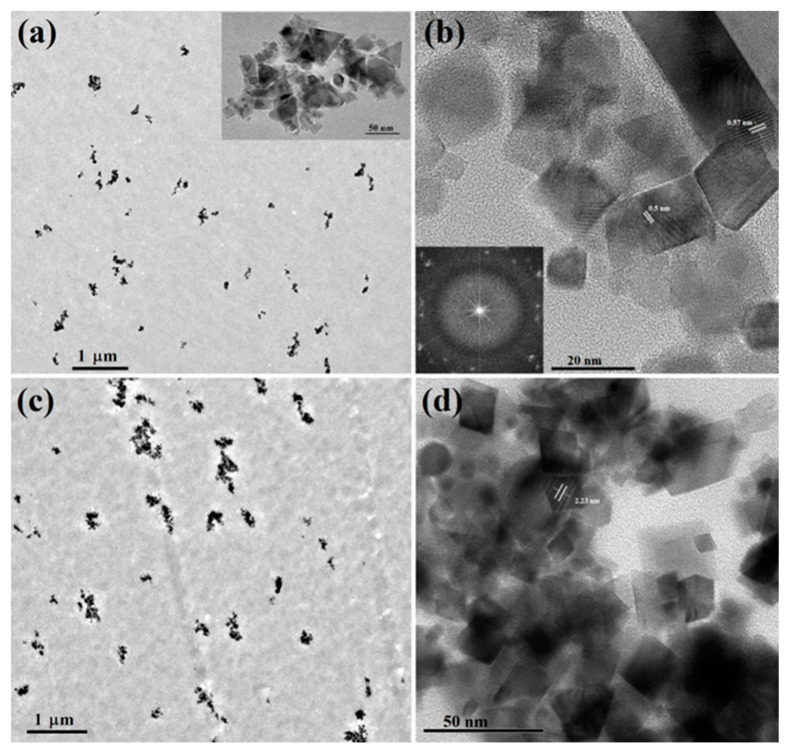
Transmission electron microscopy (EM) images of CACe-1 (**a**,**b**) and CACe-5 (**c**,**d**) nanocomposites. The inset of image (**b**) shows the corresponding SAED pattern.

Further, the presence of CeO_2_ NPs in the cellulose films was confirmed using TEM analysis shown in Figure 6. As can be observed for CACe-1 (Figure 6a) and CACe-5 (Figure 6c) films, the CeO_2_ NPs are quite uniformly dispersed in the organic phase, but they had some tendency to form small aggregates. The aggregation tendency is more obvious for the CACe-5 hybrid composite which contains a higher content of CeO_2_ NPs. TheCeO_2_ nanoparticles introduced in the cellulose acetate films have different shapes (cubic, triangular, rhomboid, parallelepiped—see Figure 6a—inset, Figure 6b,d) and sizes varying between 10 and 50 nm. The crystalline structure of the particles is highlighted by the diffraction rings of selected area electron diffraction (SAED) pattern shown in the inset of Figure 6b and also by the visualization of the lattice fringes in Figure 6b,d. The lattice fringe spacing is around 0.5 nm CeO_2_ NPs of cubic shape and 2.2 nm for the rhomboid ones.

### 3.2. Mechanical Properties and Thermogravimetric Analysis (TGA)

**Figure 7:**
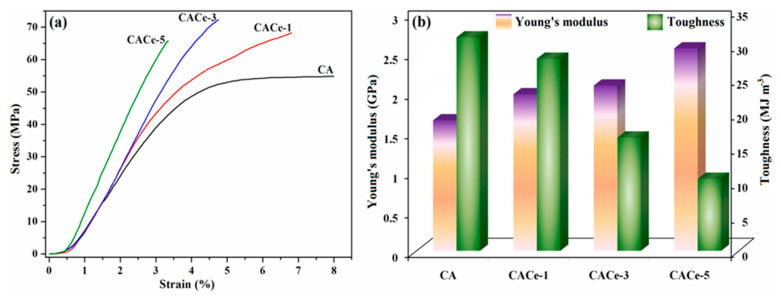
Stress–strain curves recorded for CA samples with and without CeO_2_ NPs (**a**) and the variation of Young’s modulus and toughness in the investigated composite series (**b**).

Among other favorable features, a cellulose acetate polymer is characterized by a high flexibility and good mechanical strength [34], advantageous properties from an applicability viewpoint; consequently, the mechanical performance of the pristine CA films and of the hybrid composites was comparatively analyzed. Therefore, for the prepared samples, the maximum tensile strength, elongation at break, Young’s modulus and toughness were determined; the achieved results are illustrated in Figure 7 or included in Table 1.

The stress–strain curves (Figure 7a) showed that the maximum tensile stress increases as the number of CeO_2_ NPs in the matrix enhances, from 54.9 MPa for CA to 72.3 MPa for CACe-3, while for the CACe-5 sample, the presence of 5 wt.% CeO_2_ NPs determine a decrease in the tensile strength. The improvement of tensile strength with the addition of inorganic nanoparticles can be attributed to a reinforcement effect exerted by them. However, over a certain limit (in our case above 3 wt.%), the presence of CeO_2_ NPs negatively influences the mechanical properties. Likewise, the elongation at break of the investigated films varied from 8% (CA) to 3.5% (CACe-5), suggesting that the inclusion of increasing numbers of nanoparticles provoked an augmentation in the stiffness of the hybrid films. The Young’s modulus values of the investigated samples (Figure 7b) increased gradually by enhancing the CeO_2_ content and varied between 1.65 GPa (CA) and 2.56 GPa (CACe-5) indicating a higher resistance to deformation and breakage, while the tensile toughness (measured as the area under the stress–strain curve) varied from 31 (CA) to 10.5 MJ m^−3^ (CACe-5), confirming the formation of materials more susceptible to breakage. The attained results are comparable to other cellulose acetate/nanoparticle systems reported in the literature [35,36].

**Figure 8:**
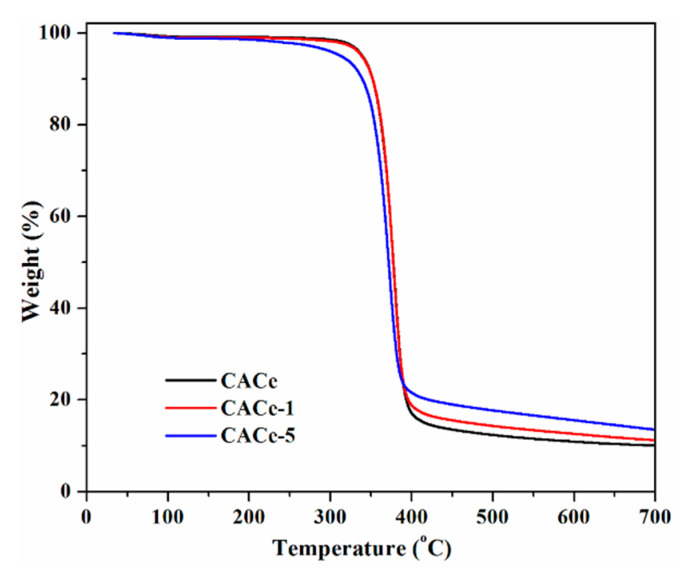
Thermogravimetric analysis (TGA) thermograms for CACe, CACe-1 and CACe-5 samples.

The results of the TGA analysis for CACe, CACe-1 and CACe-5 samples are shown in Figure 8. As can be observed, the thermal decomposition of all samples proceeds in a single step and the onset temperatures are higher than 300 °C, suggesting a good thermal stability of the investigated materials, behavior comparable to other similar systems reported in the literature [37,38]. The char residue is above 10% at 700 °C and proportionally increases with the number of inorganic nanoparticles.

### 3.3. UV Shielding Ability of CACe Nanocomposites

**Figure 9:**
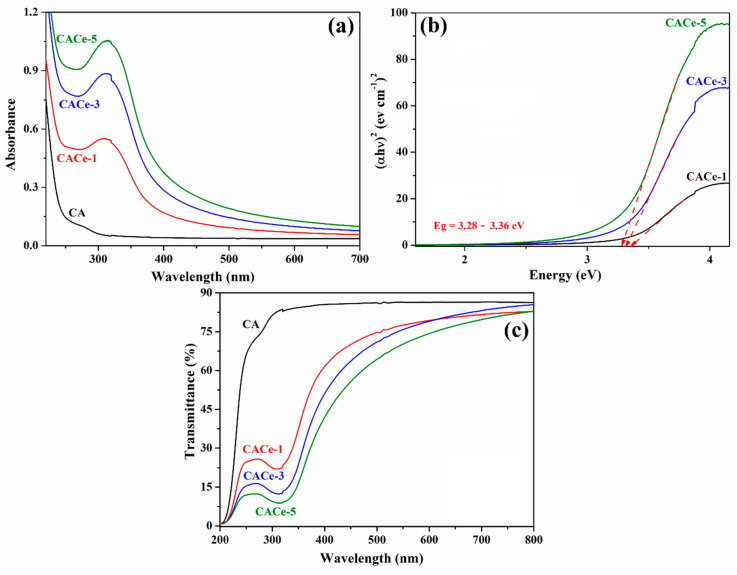
UV-Vis absorption spectra (**a**), Tauc plots (**b**) and transmittance spectra (**c**) of cellulosic films.

The optical properties of CA and CA–CeO_2_ nanocomposite films were investigated through UV-Vis spectroscopy, by measuring their UV-Vis absorption and transmittance spectra (Figure 9). It can be observed that the cellulose acetate film has no absorption neither in UV nor visible regions, at a wavelength ranging from 200 to 700 nm, meanwhile the CA films incorporating functionalized CeO_2_ NPs showed intense absorption bands in the UV domain located at around 312–317 nm (Figure 9a), due to the presence of CeO_2_ NPs in the matrix. So, these materials can be used in different fields as efficient UV absorbers, mainly for the UV-B radiation from the sunlight (280–315 nm), but also for UV-A radiation (315–400 nm). It can be observed that the intensity of absorptions peaks increases with the increase in the CeO_2_ NPs content in the cellulose acetate films (from 1 to 5 wt.%), indicating a higher UV absorption ability for CACe-5 film compared to the CaCe-3 and CaCe-1. Additionally, the optical band gap energy for the electron transition from the valence band to the conduction band of CACe hybrid films was calculated using Tauc’s Equation [39,40,41]:

(1) $${\left(\alpha h\upsilon \right)}^{2}=k\;(h\upsilon -\mathrm{Eg}),$$

where *α* is the absorption coefficient, *hυ* is the absorption energy (*h*—Planck constant, *υ*—incident frequency), k is the band tailing parameter and Eg is band gap energy. The value of the optical energy band gap was determined by the plot of ${\left(\alpha h\upsilon \right)}^{2\;}$ versus *hυ* by extrapolating the straight line until the intercept with *hυ* axis, as represented in Figure 9b. According to Figure 9b the band gap energy decreases with the rising content of CeO_2_ NPs immobilized in the matrix, being reduced from Eg = 3.36 eV for CACe-1 to Eg = 3.28 eV for CaCe-5 film. Even though the result is not spectacular, the band gap energy narrowing makes these materials suitable for UV filtering applications. Our results are comparable with those reported by W. Wang et al. [14] on regenerated cellulose films containing CeO_2_ NPs. We calculated Urbach energy which can be related to the crystalline regions in the samples or structural disorder in polymer composites, as already reported in the literature [42,43,44]. The Urbach energy is calculated with the equation [45,46]:

*α* = *α*_0_·exp(*h*ν/E_u_) (2)

where *α* is the absorption coefficient, *h*ν is the photon energy and E_u_ is the Urbach energy. The Urbach energy values were determined as the reciprocal of the slope of the linear fit of the plots in(*α*) versus E, below the optical band gap region. The absorption coefficient α is proportional to absorbance F(R), and consequently we have plotted in(F(R)) versus the E [47]. The calculated Urbach energy was found to be E_u_ = 125 meV for the CACe-1 film and it decreased to E_u_ = 101 meV for CACe-5 composite. The decrease in this parameter indicates an increase in the crystalline region in the films in the order CACe-1 < CACe-3 < CACe-5, which is attributed to the increase in CeO_2_ NPs content in the cellulosic films. Furthermore, the E_u_ value decrease from CACe-1 tot CACe-5 indicates a diminution of the structural disorder in polymer composites with the growth of CeO_2_ NPs content [42].

Moreover, the transmittance spectra of the CA and CA-CeO_2_ nanocomposite films showed in Figure 9c confirm the potential use of our materials for the above mentioned applications. The CA film is transparent to UV (from 250 nm) and visible light, the transmittance value at 600 nm is 85%, meanwhile the CACe films block the UV light, the absorption degree depending on the CeO_2_ NPs loading in the films. At 350 nm, CACe-1 absorbs 63% of UV-A radiation, CACe-3 blocks 77% of UV-A radiation and CACe-5 absorbs 84% of UV-A radiation. Beyond the UV protection characteristic, the CACe films are also transparent to the visible light, the transmittance values at 600 nm are as follows: 79.3% for CACe-1, 79.0% for CACe-3 and 74.3% for CACe-5. Our films have similar UV-shielding properties with other materials reported in the literature such as TiO_2_/cellulose acetate hybrid films [36] or poly(L-lactide)/ZnO nanocomposites [48]. All these results recommend our materials as promising candidates for UV filtering application, mainly as UV-protective coatings in food packing, especially considering the current trends of the replacement of classic materials derived from petroleum (e.g., polyethylene) with renewable and biodegradable ones (cellulose derivatives) [49,50] in order to offer green solutions for environment protection.

**Figure 10:**
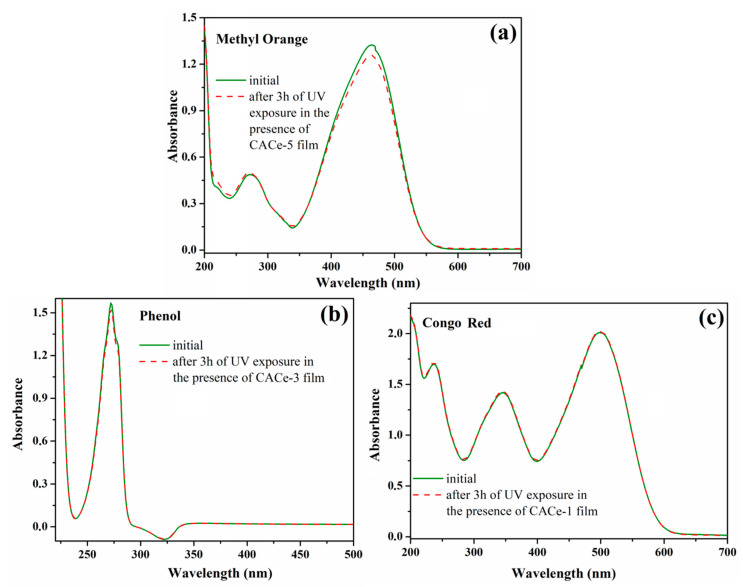
Changes of UV-Vis absorption spectra of aqueous Methyl Orange (**a**), phenol (**b**) and Congo Red (**c**) solutions in the presence of CA-CeO_2_ films used as catalysts monitored after 3 h of UV irradiation exposure.

Another very important aspect to consider for the materials used in the food sector is their inertness under sunlight and to external chemicals, avoiding thus toxic reactive oxygen species (ROS) (hydroxide, hydrogen peroxide, nitroxyl) formation which can cause lipids oxidation and implicitly food quality deterioration (e.g., off-odors, off-flavors, texture and color changes, nutrition losses) [51]. In CeO_2_, both Ce^3+^ and Ce^4+^ oxidation states coexist inducing deactivation of toxic ROS as well as the UV absorption property, and the photocatalytic activity of CeO_2_ is strongly reduced by the system oxygen deficiency [52,53]. Consequently, in the next experiment we have demonstrated the lack of photocatalytic activity of our materials and their participation to ROS generation. The photocatalytic activities of CACe-1—5 films were tested on the photodecomposition of some model pollutants such as Methyl Orange, phenol and Congo Red. The experiments were realized by immersing the hybrid films containing CeO_2_ NPs in the pollutants’ aqueous solutions and exposed them in ambient conditions to UV irradiation, with intensity at the same value of that from solar light (ca. 8 mW/cm^2^) for 3 h. The photodecomposition process of the pollutants was followed through UV-Vis spectroscopy by monitoring their characteristic absorption bands before and after exposure to UV light in the presence of CACe film. As can be noticed from Figure 10, the degradation process does not take place for any of the investigated compounds in the presence of our materials. Hence, the cellulose–CeO_2_ nanocomposite films do not possess photocatalytic activity under UV irradiation and therefore do not generate ROS causing toxicity, this being another point that endorses them to be used as UV-protective coatings in food packing.

## 4. Conclusions

This study reports the preparation and characterization of hybrid nanocomposite films based on cellulose acetate and different numbers of aminosilane-immobilized CeO_2_ NPs. Firstly, the surface of cerium oxide nanoparticles was functionalized with 3-aminopropyl (diethoxy)methylsilane as a modifying agent, and further cellulose acetate/CeO_2_ nanocomposite films were achieved via solution casting method. The prepared samples were investigated by FTIR, EDX, SEM, TEM, TGA and UV-visible spectroscopy. The structural and morphological characterization of the hybrid films confirmed the interactions among the two phases as well as the uniform distribution of the inorganic component in the cellulosic material, while the mechanical testing showed an improvement in the tensile strength with the addition of inorganic nanoparticles (up to 3 wt.%) due to the strengthening effect exerted by CeO_2_ NPs. The investigation of the optical properties of nanocomposite films revealed that these films exhibit intense absorption bands in the UV domain (around 312–317 nm) attributed to CeO_2_ NPs; consequently, they can be successfully used as efficient UV absorbers, especially as UV-protective coatings in food packing. Furthermore, the lack of photocatalytic activity under UV irradiation for cellulose–CeO_2_ nanocomposites does not determine the generation of ROS that may cause toxicity and food quality deterioration, another advantage that supports the use of these products as materials for the food packaging industry.

## Figures and Tables

**Table 1:** Mechanical parameters for the investigated films.

Sample	Maximum Tensile Strength (MPa)	Elongation at Break (%)	Young’s Modulus (GPa)	Toughness (MJ m^−3^)
CA	54.9	8	1.65	31.03
CACe-1	68.2	6.82	1.98	27.92
CACe-3	72.3	4.75	2.09	16.46
CACe-5	65.7	3.5	2.56	10.47
